# Age-related microRNAs in older breast cancer patients: biomarker potential and evolution during adjuvant chemotherapy

**DOI:** 10.1186/s12885-018-4920-6

**Published:** 2018-10-22

**Authors:** Bruna Dalmasso, Sigrid Hatse, Barbara Brouwers, Annouschka Laenen, Lieze Berben, Cindy Kenis, Ann Smeets, Patrick Neven, Patrick Schöffski, Hans Wildiers

**Affiliations:** 1 Department of Oncology, Laboratory of Experimental Oncology (LEO), Leuven, KU Belgium; 2 Department of General Medical Oncology, University Hospitals Leuven, Leuven Cancer Institute, Leuven, Belgium; 3 Interuniversity Centre for Biostatistics and Statistical Bioinformatics, Leuven, Belgium; 4 Department of General Medical Oncology and Geriatric Medicine, University Hospitals Leuven, Leuven, Belgium; 5 Multidisciplinary Breast Center, University Hospitals Leuven, Leuven, Belgium; 6 Genetics of Rare Cancers, Department of Internal Medicine and Medical Specialties, University of Genoa, Genoa, Italy

**Keywords:** Breast cancer, microRNA, Aging, Elderly, Adjuvant chemotherapy, Biomarkers, Oncogeriatrics

## Abstract

**Background:**

MicroRNAs (miRNAs) are important regulators of cellular function and have been associated with both aging and cancer, but the impact of chemotherapy on age-related miRNAs has barely been studied.

Our aim was to examine whether chemotherapy accelerates the aging process in elderly breast cancer patients using miRNA expression profiling.

**Methods:**

We monitored age-related miRNAs in blood of women, aged 70 or older, receiving adjuvant chemotherapy (docetaxel and cyclophosphamide, TC) for invasive breast cancer (chemo group, CTG, *n* = 46). A control group of older breast cancer patients without chemotherapy was included for comparison (control group, CG, *n* = 43). All patients underwent geriatric assessment at inclusion (T0), after 3 months (T1) and 1 year (T2). Moreover, we analysed the serum expression of nine age-related miRNAs (miR-20a, miR-30b, miR-34a, miR-106b, miR-191, miR-301a, miR-320b, miR-374a, miR-378a) at each timepoint.

**Results:**

Except for miR-106b, which behaved slightly different in CTG compared to CG, all miRNAs showed moderate fluctuations during the study course with no significant differences between groups. Several age-related miRNAs correlated with clinical frailty (miR-106b, miR-191, miR-301a, miR-320b, miR-374a), as well as with other biomarkers of aging, particularly Interleukin-6 (IL-6) and Monocyte Chemoattractant Protein-1 (MCP-1) (miR-106b, miR-301a, miR-374a-5p, miR-378a-3p). Moreover, based on their ‘aging miRNA’ profiles, patients clustered into two distinct groups exhibiting significantly different results for several biological/clinical aging parameters.

**Conclusions:**

These results further corroborate our earlier report, stating that adjuvant TC chemotherapy does not significantly boost aging progression in elderly breast cancer patients. Our findings also endorsed specific age-related miRNAs as promising aging/frailty biomarkers in oncogeriatric populations.

**Trial registration:**

ClinicalTrials.gov, NCT00849758. Registered on 20 February 2009. This clinical trial was registered prospectively.

## Background

(Breast) cancer treatment in the elderly represents a major challenge in clinical oncology. Despite their important representation within the overall breast cancer (BC) population, older women are often excluded from standard BC medical treatment regimens, or are offered less aggressive (and possibly less effective) therapies. This is attributable to concerns about increased risk of side effects, as well as decreased medical fitness of older cancer patients, either perceived or assumed by subjective clinical evaluation. According to international guidelines for patient management in geriatric oncology, it is not justified to base treatment choices on chronological age only [1]. In addition to the already available geriatric assessment (GA) tools, new clinical and biological tools, including circulating biomarkers of aging, are currently being developed to better assess global health and individualize therapeutic strategies [2–8].

Besides the risks that chemotherapy imposes to frail patients, the impact that it may have on the aging process of more fit elderly patients is still not well understood. Based on the observation of cellular senescence induced by cytotoxic agents (including anticancer molecules) [9], researchers have been investigating possible effects of chemotherapy on aging progression. Data obtained from follow-up studies have shown that adult survivors of childhood cancers develop degenerative diseases (typical of old age) earlier in life and with a higher incidence compared to the general population [10]. Chemotherapy-induced aging and frailty are assumed to be caused, among other factors, by production of free radical intermediates, persistent DNA damage not counterbalanced by adequate DNA repair mechanisms, and/or senescence triggered by telomere instability [11]. In a small cohort of patients with head and neck cancer, telomeres of peripheral blood mononuclear cells were reported to be severely shortened after combined chemo-radiotherapy, especially in older patients [12]. Similar results were also obtained in patients who underwent chemotherapy for BC [13] and non-Hodgkin lymphoma [14].

To investigate the hypothesized acceleration of the aging process by cancer treatment we have recently conducted a prospective clinical study in older (70+) BC patients, that monitored the evolution during adjuvant chemotherapy of both clinical aging parameters (GA) and various biological markers described in the literature as potential biomarkers of aging. This biomarker panel included leukocyte telomere length, the inflammation-related plasma cytokines/chemokines IL-6, Tumor Necrosis Factor-alpha (TNFα), Interleukin-10 (IL-10), Regulated on Activation, Normal T-Cell Expressed and Secreted (RANTES)/ C-C motif chemokine-5 (CCL5) and MCP-1/C-C motif chemokine-2 (CCL2) as markers of ‘inflammaging’, and the aging-related protein Insulin-like Growth Factor-1 (IGF-1) [5, 15–23].

Clinical and biomarker assessments were accomplished at three different time points, i.e. prior to initiation of chemotherapy, after 3 months (last cycle of chemotherapy) and after 1 year. The study comprised a cohort of BC patients receiving docetaxel/cyclophosphamide treatment and a control cohort receiving only hormone treatment. The results of the above-mentioned work have been published recently [24].

In the present study, we attempted to further expand our insight into the potential connections between adjuvant chemotherapy and biological aging, by analysing the behaviour of circulating aging-related miRNAs previously identified by our group [25].

Indeed, in addition to the above-mentioned aging biomarkers, several miRNAs have also been implicated in the aging process [25, 26]. MiRNAs are small molecules (18–22 nucleotides) belonging to the wider class of non-coding RNAs, which exert non-permanent epigenetic functions via the post-transcriptional regulation of gene expression [27]. Mutations and/or altered expression of miRNAs have been associated with several pathological conditions, including cancer [28–40]. Moreover, miRNAs may play a role in the aging process, as some of them are part of molecular pathways that regulate cellular senescence. The expression of miR-34a, for instance, is induced by p53, and high levels of this miRNA result in growth arrest [41, 42]. This miRNA has also been positively associated with myocardial aging. Its primary target is the longevity-associated deacetylase SIRT-1 [43] that regulates chromatin remodelling, stress responses, DNA repair and insulin regulation.

Several of the miRNAs reported in the literature to be associated with aging have also been found to be over- or underexpressed in cancer patients [29, 34, 35, 39, 40]. To identify plasma miRNAs with aging biomarker potential within a cancer population, we have first carried out a plasma miRNA screening study involving young and older BC patients [25]. From this pilot study, we have established a panel of miRNAs that were found to be differentially expressed according to age among BC patients: miR-20a, miR-30b, miR-34a, miR-106b, miR-191, miR-301a, miR-320b, miR374aand miR-378a-3p. Here, we have examined the quantitative fluctuations of these ‘aging miRNAs’ during and after adjuvant chemotherapy.

## Methods

### Patient population

From 2009 to 2012, women at least 70 years old who were affected by locally-advanced, non-metastatic BC and eligible for adjuvant systemic chemotherapy were enrolled at 5 hospitals in Belgium, henceforth referred to as “chemotherapy group” (CTG). This group consisted originally of 57 patients, and miRNA data were available at the 3 time points in 46 patients.

In parallel, a comparable series of patients, not eligible for systemic chemotherapy, but only for endocrine treatment were included as a “control group” (CG) [24]. This group consisted originally of 52 patients, and miRNA data were available at the 3 time points in 43 patients.

Patient and tumor characteristics of the study cohort can be found in Table 1 of our previous publication [24].

**Table 1:** Evolution of microRNAs over time in CTG

MicroRNA^a^	Inclusion versus 3 months			Inclusion versus 1 year			Study arm by time interaction^c^
	Mean difference^b^	95%CI	*p*-value	Mean difference^b^	95%CI	*p*-value	*p*-value
miR-20a	−0.52	(−1.05, 0.01)	0.0537	−0.22	(−0.75, 0.31)	0.4042	0.0898
miR-30b	−0.39	(−0.60, − 0.18)	**0.0003**	−0.22	(− 0.42, − 0.02)	**0.0319**	0.0987
miR-34a	0.76	(0.25, 1.26)	**0.0039**	0.60	(0.18, 1.01)	**0.0054**	0.9884
miR-106b	0.03	(−0.17, 0.23)	0.7671	0.18	(0.00, 0.37)	**0.0497**	**0.0240**
miR-191	−0.09	(− 0.32, 0.15)	0.4669	− 0.27	(− 0.51, − 0.03)	**0.0272**	0.2045
miR-301a	− 0.11	(− 0.30, 0.09)	0.2673	−0.15	(− 0.39, 0.09)	0.2280	0.6222
miR-320b	0.10	(−0.15, 0.34)	0.4377	−0.01	(−0.22, 0.20)	0.9337	0.4120
miR-374a	−0.28	(−0.55, − 0.01)	**0.0394**	−0.12	(− 0.38, 0.13)	0.3470	0.2125
miR-378a	−0.02	(−0.21, 0.17)	0.8168	0.01	(−0.15, 0.16)	0.9413	0.6858

^a^miR-34a, miR-320b and miR-378a were previously shown to increase with aging; miR-20a, miR-30b, miR-106b, miR-191, miR-301a and miR-374a were previously shown to decrease with aging

^b^miRNA normalized relative quantities were log2-transformed prior to statistical analysis; log2 values were subtracted to calculate mean differences between time points

^c^Significant interaction indicates different miRNA evolution in CTG as compared to CG

*p*-values < 0.05 are marked in bold

The antineoplastic therapy administered to the CTG consisted of docetaxel at a dose of 75 mg/m^2^ plus cyclophosphamide at 600 mg/m^2^ every 3 weeks for a total of 4 cycles (TC scheme) [44] . G-CSF (granulocyte-colony stimulating factor) was administered at each cycle, according to the National Comprehensive Cancer Network (NCCN) guidelines [45]. The planned adjuvant treatment also included an aromatase inhibitor (to be administered after chemotherapy completion) in case of hormone-sensitive tumors, and trastuzumab administration in case of Her2 positive tumors.

Conversely, patients in the CG received an aromatase inhibitor as sole medical treatment. In both groups, radiation therapy was either or not administered according to institutional practice. Enrolment took place after breast surgery. Blood samples were collected at three time points: T0: between 3 and 6 weeks after surgery, always before the first cycle of chemotherapy; T1: 3 months after inclusion (in principle the day of the fourth and last cycle of chemotherapy for patients in the CTG); T2: 1 year after inclusion. At each time point, patients also underwent clinical geriatric evaluation.

This study was approved by the local ethics committees of Clinique Sainte Elisabeth (Namur), Imelda Hospital (Bonheiden), Jessa Hospital (Hasselt), Zol Ziekenhuis Oost-Limburg (Genk), Jules Bordet Institute (Brussels), and by the University Hospitals Leuven central ethics committee. All patients signed a written informed consent in accordance to the Helsinki Declaration. This clinical trial was registered prospectively.

This article adheres to CONSORT guidelines, where applicable.

### Clinical geriatric evaluation

Detailed information on GA tools and results of clinical evaluation accomplished at the different time points have been extensively documented in our primary study on aging biomarkers and chemotherapy [24]. Briefly, patients were screened at baseline with the G8 screening tool and the Flemish version of the Triage Risk Screening Tool (fTRST), and social data were collected (age, living situation, marital status and educational level).

At each time point also a geriatric assessment (GA) was performed, as well as a frailty assessment with Balducci Frailty score and Leuven Oncogeriatric Frailty Score (LOFS) [5]. Moreover, we assessed performance status according to the Eastern Cooperative Oncology Group - Performance Status (ECOG-PS), and quality of life (QoL) using the EORTC QLQ-C30 questionnaire.

At both T1 and T2, adverse events (using the CTCAE v4.0 classification) and unexpected hospitalizations were also monitored.

### Endpoints

Our primary endpoint was to evaluate whether aging-related miRNAs changed during the study period and, if so, whether relevant differences could be detected over time between CTG and CG.

As secondary endpoints, we also assessed (i) potential correlations of miRNAs measured at inclusion (T0) with chronological age, clinical geriatric assessment parameters and aging biomarkers reported in our primary paper [24]; (ii) whether miRNAs at inclusion had a predictive value towards acute and/or irreversible decline in functionality and in QoL; (iii) whether miRNAs at inclusion predicted toxicity and unexpected hospitalizations during and after chemotherapy; (iv) correlation patterns between the 9 miRNAs and possible formation of patient clusters based on miRNA expression profiles.

### Blood sample collection and processing

At each time point, 4-mL whole blood specimens were collected from each patient in BD Vacutainer SST II Advance serum tubes. After incubation at room temperature for 20 to 60 min, the blood samples were centrifuged at 1300×g for 10 min at 4 °C and supernatants (serum) were aliquoted and stored at − 80 °C.

### Aging biomarker analysis

Methodology and results of biomarker analyses (i.e. leukocyte telomere length, circulating IL-6, IL-10, TNFα, RANTES/CCL5, MCP-1/CCL2 and IGF-1) performed at the 3 time points were described in detail in our previous publication [24].

### Isolation of miRNAs from serum

For each sample, 250 μL of serum were thawed on ice and then centrifuged at 3000 x g for 5 min to remove debris. To 200 μL of supernatant, 1 μg of carrier MS2 RNA (Roche) was added in order to stabilize RNA during extraction and cDNA synthesis. Moreover, 1 μL of the synthetic RNA spike-in UniSp6 was added to allow evaluation of the efficiency and uniformity of the entire RNA extraction/cDNA synthesis procedure. Then, miRNAs were isolated with the miRCURY™ RNA Isolation Kit–Biofluids (Exiqon), following the manufacturer’s instructions. Spin columns were finally eluted twice with 25 μL DNase/RNase-free water each time. Both eluates were pooled and stored at − 80 °C.

### cDNA synthesis and qPCR

On each purified miRNA sample, cDNA synthesis was performed in duplicate. 2 μL of miRNA extract were processed using the Universal cDNA synthesis kit II (Exiqon), according to the manufacturer’s instructions. cDNA samples were stored at − 80 °C until PCR analysis.

Measurement of relative amounts of transcripts was carried out by real-time qPCR analysis using Pick-&-Mix microRNA PCR panels (96 well Ready-to-Use custom plates) with Exilent SYBR® Green Master Mix (Exiqon). For each RNA sample, both duplicate cDNAs were assessed in a single plate. Every plate included primers for: 5 reference miRNAs (hsa-miR-23a-3p, hsa-miR-29a-3p, hsa-miR-29c-3p, hsa-miR-140-3p, hsa-miR-484, further referred to as miR-23a, miR-29a, miR-29c, miR-140 and miR-484) used for data normalization, the 9 aging-related miRNAs of interest selected for the study (see below) and the synthetic spike-in UniSp6 to allow evaluation of miRNA extraction/reverse transcription efficiency. The 9 aging miRNAs included 3 that were previously shown to increase with aging (hsa-miR-34a-5p, hsa-miR-320b and hsa-miR-378a-3p, further referred to as miR-34a, miR-320b and miR-378a), and 6 that previously showed negative association with aging (hsa-miR-20a-3p, hsa-miR-30b-5p, hsa-miR-106b-5p, hsa-miR-191-5p, hsa-miR-301a-3p and hsa-miR-374a-5p, further referred to as miR-20a, miR-30b, miR-106b, miR-191, miR-301a and miR-374a) [25]. All plates additionally included the interplate calibrator UniSp3, in order to allow detection of global amplification differences due to inter-run variation.

Assays were carried out following the manufacturer’s specifications. Briefly, cDNA was diluted 50x in nuclease-free water and mixed with an equal volume of 2x Exilent SYBR Green master mix (Exiqon). Final reaction volume was 10 μL. Plates were run on a LightCycler 480 (LC480, Roche) instrument applying the following thermal cycling protocol: activation step (10 min at 95 °C); 45 amplification cycles (10 s at 95 °C, 1 min at 60 °C, ramp rate 1,6 °C/s); melting curve analysis.

### Quality control and processing of PCR data

As haemolysis can alter the relative amounts of different serum miRNAs through the release of intracellular miRNAs from erythrocytes, a quality control was performed using the miR-451/miR-23a-3p hemolysis test [46]. Following qPCR analysis of the expression of miR-451 (highly expressed in erythrocytes) and miR-23a-3p (stably expessed in biofluids), all samples with a Delta Cp value (Cp miR-451 minus Cp miR-23a-3p) higher than 5 were excluded from further analysis. Samples with borderline results (Delta Cp between 4 and 5) were double-checked for haemolysis using a second method. Thawed serum samples were briefly spun down to remove debris, and then the absorbance spectrum was assessed on a Nanodrop ND-1000. Samples showing an apparent absorption peak at 415 nm (the hemoglobin absorption maximum) were excluded from the study. The qPCR data were processed using the MultiD GenEx software. We visually inspected expression profiles of all miRNAs and the UniSp6 across all samples on a bidimensional line plot. Samples with a clearly deviating expression for the entire miRNA panel were excluded from further analysis. Normalization was performed using the 5 reference transcripts miR-23a-3p, miR-29a-3p, miR-29c-3p, miR-140-3p and miR-484. These were validated in our previous paper [25] as suitable reference miRNAs for serum/plasma samples by the algorithm tools GeNorm and NormFinder and were now again confirmed to be stably expressed across all serum samples. Technical repeats (duplicate cDNAs per sample) were averaged and finally, all values were converted to relative quantities and then log-transformed (Log2 scale).

### Statistical analysis

For the primary endpoint, miRNAs were modelled as response variables in linear models for repeated measures with time, group and their interaction as explanatory variables. An unstructured residual covariance matrix was modelled to account for clustering.

The evolution over time in the CTG was assessed by estimating the change in miRNA level between inclusion (T0) and 3 months (T1) and between inclusion (T0) and 12 months (T2). Results were presented by the mean change between time points with 95% confidence interval (CI). The difference in evolution between chemotherapy and control patients was assessed by a test for group by time interaction.

For the secondary endpoints, Spearman correlations were used for studying univariable association of miRNAs with continuous or ordinal variables. Kruskal-Wallis tests were used to compare miRNA levels between more than 2 groups, and Mann-Whitney U tests for comparisons between two groups. Multivariable models: a backward selection procedure was applied for selecting a set of miRNA as independent predictors of response variables (age, clinical aging parameters and aging biomarkers). Linear regression was used for continuous variables, logistic regression for binary variables, and proportional odds models for ordinal variables. Mann-Whitney U tests were used for comparing miRNA levels between patients with and without decline in functionality, unexpected hospitalization or grade II-III-IV toxicity.

The association between the miRNAs were studied by means of Pearson correlations. To identify groups (clusters) of patients with similar miRNA profiles, a disjoint cluster analysis was performed based on minimizing the sum of squared (euclidian) distances from the cluster means; miRNA values were standardized for this analysis. To decide upon the number of clusters, we took into account the pseudo F statistic (larger means better fit) and the number of patients per cluster. The SAS procedure FASTCLUS was used for this analysis. Analyses were performed for data measured at inclusion. Mann-Whitney U tests were used for comparing patients within two clusters on ordinal or continuous variables. Fisher exact tests were used for comparing clusters on categorical or binary outcomes (decline, hospitalization, toxicity).

All tests were two sided, and a 5% significance level was considered for all tests.

All analyses have been performed using SAS software, version 9.4 of the SAS System for Windows. Copyright © 2002 SAS Institute Inc. SAS and all other SAS Institute Inc. product or service names are registered trademarks or trademarks of SAS Institute Inc., Cary, NC, USA.

Figures were performed using using GraphPad Prism version 6.00 for Windows, GraphPad Software, La Jolla, CA, USA.

## Results

### Evolution of aging miRNAs over time during BC treatment

**Fig. 1:**
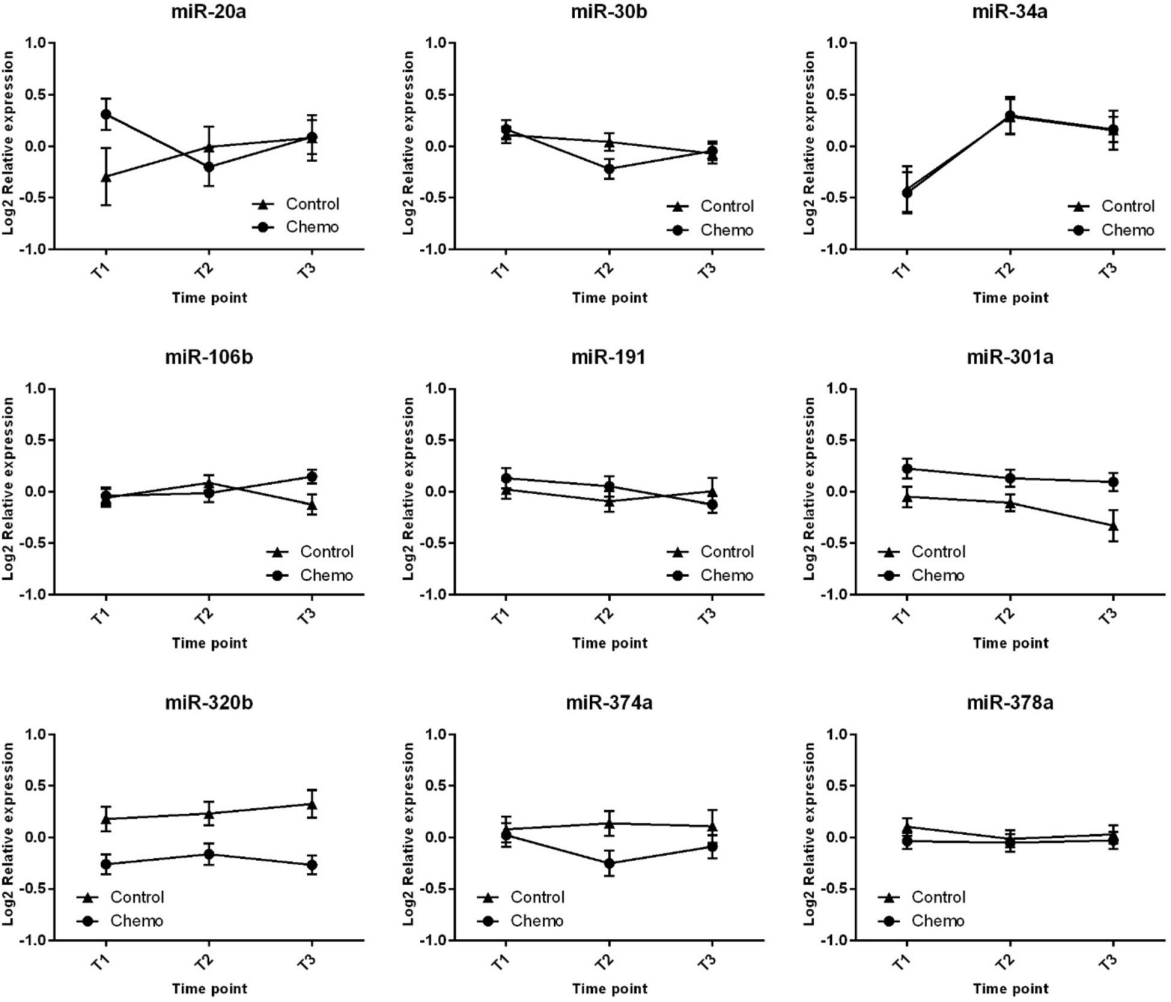
Time evolution of aging miRNAs in ChG and CoG

For each miRNA, time evolution in both CTG and CG is shown in Fig. 1 and the corresponding statistics are summarized in Table 1. Several miRNAs showed significant changes in patients of CTG during the course of the study: miR-34a was increased at T1 (*p* = 0.0039) while miR-30b and miR-374a were decreased at T1 (*p* = 0.0003 and 0.0394, respectively). For miR-374a, these changes appeared to be transient: the initial miRNA levels measured at inclusion (T0) were restored after 1 year (T2). In contrast, the observed changes of miR-30b and miR34a still persisted after 1 year, albeit somewhat less pronounced (mean differences were − 0.22 at T2 versus − 0.39 at T1 for miR-30b and 0.60 at T2 versus 0.76 at T1 for miR-34a). However, for none of these three miRNAs, a significant difference in evolution over time could be demonstrated when comparing CTG with CG, as indicated by the lack of a statistically significant group by time interaction. Plasma levels of miR-106b and miR-191 were found to be slightly increased (*p* = 0.0497), respectively decreased (*p* = 0.0272) in CTG at T2 but not at T1. Moreover, a significant group by time interaction (*p* = 0.024) for miR-106b may point to a different evolution of this miRNA in CTG versus CG. No significant modifications were observed for the other miRNAs (miR-20a, miR-301a, miR-320b, miR-374a, miR-378a) during the time course of the study (Fig. 1**,** Table 1). Overall, the observed differences over time did not appear to depend on the type of administered treatment, hinting toward the lack of an effect of chemotherapy on aging in the analysed population. Of note, 3 miRNAs (miR-20a, miR-301a, miR-320b) were significantly different at baseline (in the direction of increased aging) in CG compared to CTG, corresponding to the fact that clinical aging was also slightly more pronounced in CG (see Table 3 from our recent publication [24]).

### Association of aging miRNAs with patient’s chronological age

In initial univariable analyses, patient age at inclusion, strongly tended to be associated with several previously identified aging related miRNAs [25] measured at T0 (i.e. miR-30b, miR-374a, miR-106b, miR-301a, miR-320b), even within this ‘purely elderly population’ (Table 2).

**Table 2:** Univariable association of microRNAs with chronological age and geriatric assessment parameters at inclusion

	Calendar age		Balducci frailty score (median)				LOFS (*N* = 87)		G8 (*N* = 89)		fTRST (*N* = 66)	
Micro RNA^*a*^	Spearman correlation	*p*-value	Fit (*N* = 21)	Vulnerable (*N* = 29)	Frail (*N* = 38)	*p*-value	Spearman correlation	*p*-value	Spearman correlation	*p*-value	Spearman correlation	*p*-value
miR-20a	−0.061	0.5693	0.38	0.16	0.54	0.577	0.023	0.8304	0.037	0.7289	−0.054	0.6685
miR-30b	−0.202	0.0578	0.14	0.14	0.10	0.829	0.086	0.4306	−0.057	0.5967	−0.065	0.6059
miR-34a	0.043	0.6867	−0.41	−0.21	0.00	0.711	− 0.039	0.7224	0.015	0.8918	0.090	0.4729
miR-106b	−0.211	**0.0472**	0.07	0.01	0.09	0.837	−0.040	0.7131	−0.014	0.8966	0.004	0.9755
miR-191	−0.144	0.1771	0.13	0.06	−0.22	0.080	0.223	**0.0376**	−0.008	0.9413	−0.167	0.1798
miR-301a	−0.285	**0.0068**	0.38	0.35	−0.05	**0.037**	0.257	**0.0164**	0.024	0.8215	−0.322	**0.0084**
miR-320b	0.186	0.0802	0.06	−0.15	− 0.10	0.575	− 0.037	0.7342	− 0.076	0.4793	0.056	0.6533
miR-374a	−0.212	**0.0466**	0.11	0.10	−0.23	0.163	0.308	**0.0037**	0.185	0.0823	−0.221	0.0747
miR-378a	0.030	0.7827	0.02	0.03	0.12	0.642	−0.161	0.1359	−0.095	0.3753	0.055	0.6585

*p*-values < 0.05 are marked in bold

^a^miR-34a, miR-320b and miR-378a were previously shown to increase with aging; miR-20a, miR-30b, miR-106b, miR-191, miR-301a and miR-374a were previously shown to decrease with aging

In a next step, a backward multivariable model selection procedure was applied, resulting in a model with miR-301a as the only independent explanatory variable for age. The model revealed a negative association between miRNA-301a and age: higher age is associated with lower miR-301a levels (*p* = 0.0006) (Table 3), which is in accordance with our previous findings [25].

**Table 3:** Independent predictors of chronological age and clinical/biological aging markers at inclusion

Response variable	Independent Predictor(s) ^*c*^	Slope^*a*^ Odds Ratio^*b*^	95%CI	*p*-value
Calendar age ^*a*^	miR-301a	−2.371	(−3.700; −1.042)	0.0006
BALDUCCI ^*b*^	–	–	–	–
LOFS ^*a*^	miR-320b	− 0.623	(− 1.187; − 0.059)	0.0308
	miR-374a	0.940	(0.396; 1.483)	0.0009
G8-total ^*a*^	miR-106b	−1.145	(−2.082; − 0.209)	0.0171
	miR-191	− 1.461	(− 2.478; − 0.444)	0.0054
	miR-320b	− 0.776	(− 1.461; − 0.092)	0.0266
	miR-374a	1.209	(0.436; 1.982)	0.0026
fTRST ^*b*^	miR-301a	0.487	(0.254; 0.934)	0.0302
Telomere length ^*a*^	miR-320b	−0.095	(− 0.170; − 0.021)	0.0131
IL-6 ^*a*^	miR-106b	−0.338	(−0.603; − 0.072)	0.0133
	miR-374a	− 0.294	(− 0.497; − 0.090)	0.0053
	miR-378a	0.301	(0.011; 0.591)	0.0418
IL-10 ^*a*^	–	–	**–**	–
IGF-1 ^*a*^	–	–	–	–
TNFα ^*a*^	miR-320b	0.193	(0.040; 0.345)	0.0140
MCP-1 ^*a*^	miR-301a	−0.180	(− 0.320; − 0.040)	0.0121
	miR-378a	0.269	(0.098; 0.441)	0.0025
RANTES ^*a*^	miR-378a	−0.390	(− 0.736; − 0.045)	0.0271

^a^Continuous variable; slope indicates mean change in response variable for a 1-unit increase of miRNA values. Slope > 0 indicates positive association; slope < 0 indicates inverse correlation

^b^Ordinal variable; odds ratio > 1 indicates increase in response variable with increased miRNA value (positive association); odds ratio < 1 indicates decrease in response variable with increased miRNA value (negative association)

^c^miR-34a, miR-320b and miR-378a were previously shown to increase with aging; miR-20a, miR-30b, miR-106b, miR-191, miR-301a and miR-374a were previously shown to decrease with aging

### Association of miRNAs with clinical aging

Next, we investigated whether the ‘aging miRNAs’, measured at inclusion, correlated with the patient’s clinical aging status, also assessed at T0.

The output of the univariable analysis (Table 2) reported a positive correlation of miR-191, miR-301a and miR-374a with LOFS score. In the subsequent multivariable model (Table 3), however, miR-320b and miR-374a emerged as independent predictors for LOFS, with higher LOFS scores being associated with higher miR374a and lower miR-320b levels. Note that different statistical techniques used for univariable and multivariable analysis may account for the apparent discrepancy in miRNAs arising as significant predictors.

Conversely, none of the miRNAs showed an association with the Balducci frailty score as ordinal outcome: the preliminary observed difference of miR-301a levels between the 3 categories fit, vulnerable and frail (*p* = 0.037) (Table 2) was not confirmed in the multivariable analysis (Table 3).

Although none of the miRNAs showed a significant individual association with G8 total score (Table 2), 4 miRNAs (miR-106b, miR-191, miR-320b, and miR-374a) resulted as independent predictors of total G8 from the backward multivariable model selection procedure (Table 3). This may point out toward a coordinated set of molecular changes reflecting the parameters assessed by this score. Concerning fTRST, which returns a higher score (scale 0–6) with increasing frailty, a negative correlation was observed for miR-301a, which was confirmed by the multivariable model selection (Tables 2 and 3).

### Association of miRNAs at inclusion with other aging biomarkers

We also examined possible correlations between the ‘aging miRNAs’ and other aging biomarkers measured at T0.

Mean leukocyte telomere length (T/S ratio) was negatively correlated with miR-320b (Table 4), which was confirmed to be the only explanatory variable in the multivariable model selection (Table 3). Two other miRNAs, miR-34a and miR-106b, were borderline significant in the univariable analysis (Table 4), but were not retained in the multivariable model selection procedure (Table 3).

**Table 4:** Univariable association of microRNAs with aging biomarkers at inclusion

MicroRNA^*a*^	Telomere length	IL-6	IL-10	IGF-1	TNFα	MCP-1	RANTES
miR-20a	−0.027 (0.8250)	−0.131 (0.2208)	− 0.141 (0.1979)	−0.045 (0.6762)	− 0.101 (0.3474)	−0.045 (0.6743)	− 0.052 (0.6273)
miR-30b	−0.169 (0.1587)	− 0.268 (**0.0111**)	−0.166 (0.1291)	0.040 (0.7078)	0.049 (0.6479)	−0.168 (0.1165)	0.057 (0.5987)
miR-34a	−0.233 (0.0509)	0.057 (0.5962)	−0.134 (0.2198)	−0.104 (0.3306)	0.099 (0.3551)	0.184 (0.0835)	−0.018 (0.8669)
miR-106b	0.228 (0.0559)	−0.281 (**0.0075**)	−0.096 (0.3841)	0.118 (0.2721)	−0.310 (**0.0031**)	− 0.288 (**0.0062**)	0.174 (0.1033)
miR-191	0.025 (0.8351)	−0.304 (**0.0038**)	−0.054 (0.6222)	0.015 (0.8891)	−0.101 (0.3478)	− 0.387 (**0.0002**)	0.074 (0.4878)
miR-301a	−0.038 (0.7507)	−0.340 (**0.0011**)	− 0.070 (0.5236)	0.098 (0.3604)	− 0.208 **(0.0505)**	−0.328 **(0.0017)**	0.029 (0.7846)
miR-320b	−0.234 (**0.0492**)	0.132 (0.2166)	0.171 (0.1181)	−0.144 (0.1771)	0.396 (**0.0001**)	0.252 (**0.0171**)	0.040 (0.7115)
miR-374a	−0.207 (0.0830)	−0.337 (**0.0012**)	− 0.248 (**0.0221**)	0.0003 (0.9777)	− 0.024 (0.8250)	−0.231 (**0.0294**)	0.192 (0.0713
miR-378a	0.018 (0.8842)	0.302 (**0.0040**)	0.153 (0.1620)	−0.015 0.8866)	0.151 (0.1568)	0.295 (**0.0051**)	−0.222 (**0.0365**)

In each cell are displayed the Spearman’s correlation coefficient, and according *p*-value in parentheses

*p*-values < 0.05 are marked in bold

^a^miR-34a, miR-320b and miR-378a were previously shown to increase with aging; miR-20a, miR-30b, miR-106b, miR-191, miR-301a and miR-374a were previously shown to decrease with aging

In univariable analysis, 5 miRNAs were found to be negatively correlated with IL-6, a cytokine well known to be increased during aging, particularly in frail individuals. Those were miR-30b, miR-106b, miR-191, miR301a and miR-374a. Conversely, miR-378a-3p showed a positive correlation with IL-6 (Table 4). These correlation trends are in line with our previous findings that miR-30b, miR-106b, miR-191 and miR-374 are all decreased, while miR-378a is increased, in elderly versus young patients, and with the widely documented age-related increase in plasma IL-6 levels [15, 16]. Of these 6 miRNAs that were significantly associated with IL-6, 3 resulted as significant independent predictors of IL-6 in the multivariable model: miR-106b, miR-374a and miR-378a (Table 3). Furthermore, TNF-α and MCP-1, which are also known to gradually increase in plasma during aging, showed pronounced associations with several ‘aging miRNAs’. As expected, negative associations were found for miRNAs showing decreased expression with higher age (miR-106b, miR-191, miR-301a, miR-374a) whereas positive associations were found for miRNAs showing increased expression with higher age (miR-378a, miR-320b) (Table 3). Higher miR-320b values were confirmed to be associated with higher TNF-α levels (*p* = 0.0140) in the subsequent multivariable selection model (Table 3). For MCP-1, the multivariable selection model further corroborated miR-301a (*p* = 0.0121) and miR-378a (*p* = 0.0025) as independent explanatory variables (Table 2). For the other plasma biomarkers (i.e. IL-10, RANTES, IGF-1), no consistent correlations were established in univariable and/or multivariable analyses (Tables 4 and 3).

### Association of miRNAs with adverse effects of chemotherapy: decline of functionality and QoL, unexpected hospitalization and toxicity

In CTG, none of the individual miRNAs measured at inclusion (T0) was predictive of decline in functionality or decline in QoL at 3 months (T1) or at 1 year (T2): initial miRNA levels at T0 did not significantly differ between patients who experienced a decline in functionality and/or QoL during the course of the study and patients who did not (all *p* ≥ 0.05). Also, in patients exhibiting a decline in functionality and/or QoL at 3 months or at 1 year, miRNA plasma levels at the corresponding time point were not significantly altered (all p ≥ 0.05). Moreover, miRNAs plasma level at inclusion neither predicted grade II-III-IV toxicity at 3 months, nor unexpected hospitalization during the whole time course (all *p* > 0.1) (results not shown).

### Correlations and cluster analysis of the 9 miRNAs

**Fig. 2:**
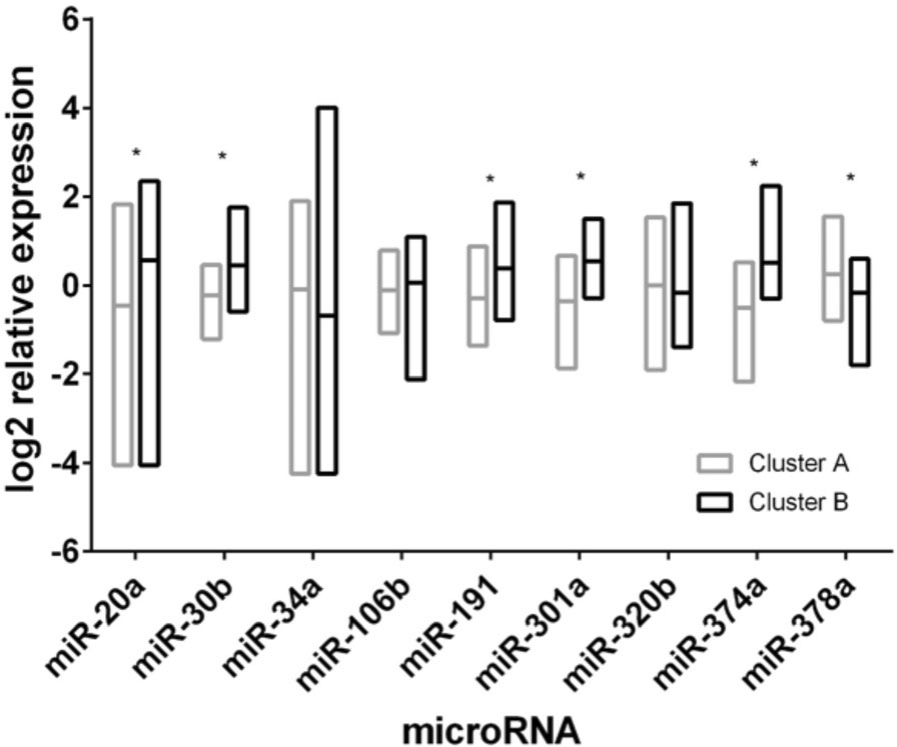
Different aging miRNA expression profile in patient clusters A and B. Significant differences (*p* < 0.001) are indicated with an asterisk

We have also examined the interrelationship between the 9 miRNAs included in the study; Table 5 summarizes Spearman’s correlation coefficients and associated *p*-values, based on miRNA measurements at inclusion. As expected, strong correlations exist between several miRNAs of the ‘aging miRNA’ panel, most particularly miR-30b, miR-191, miR-301a and miR-374a. Accordingly, a disjoint cluster analysis based on T0 miRNA measurements, revealed two main patient clusters of which one (cluster A) consistently scores lower on miR-20a, miR-30b, miR-191, miR-301a and miR-374a and higher on miR-378a compared to the other (cluster B), which is consistent with an ‘older’ aging profile for cluster A (Fig. 2). For the remaining miRNAs (miR-34a, miR106b and miR-320b), differences between patient clusters were either small or inconsistent, as shown in Fig. 2. One patient did not fit either of both clusters and was excluded from the cluster analysis.

**Table 5:** Correlation between microRNAs measured at inclusion

MicroRNA^*a*^	miR-20a	miR-30b	miR-34a	miR-106b	miR-191	miR-301a	miR-320b	miR-374a	miR-378a
miR-20a	_	0.252 (0.0174)	0.271 (0.0103)	0.225 (0.0337)	0.035 (0.7421)	0.320 (0.0022)	−0.270 (0.0106)	0.067 (0.5347)	−0.154 (0.1486)
miR-30b	.	_	−0.081 (0.4512)	−0.237 (0.0254)	**0.634 (<.0001)**	**0.624 (<.0001)**	0.352 (0.0007)	**0.722 (<.0001)**	−0.129 (0.2275)
miR-34a	.	.	_	−0.080 (0.4566)	−0.260 (0.0139)	− 0.172 (0.1070)	−0.076 (0.4766)	− 0.256 (0.0153)	−0.054 (0.6129-)
miR-106b	.	.	.	_	−0.401 (<.0001)	0.143 (0.1812)	**−0.635 (<.0001)**	−0.273 (0.0096)	− 0.106 (0.3210)
miR-191	.	.	.	.	_	**0.544 (<.0001)**	0.341 (0.0011)	**0.761 (<.0001)**	−0.235 (0.0265)
miR-301a	.	.	.	.	.	_	−0.056 (0.6036)	**0.549 (<.0001)**	−0.138 (0.1982)
miR-320b	.	.	.	.	.	.	_	0.330 (0.0016)	0.234 (0.0270)
miR-374a	.	.	.	.	.	.	.	_	−0.384 (0.0002)

Each cell displays the Spearman’s correlation coefficient, and according *p*-value in parentheses. Strong and highly significant correlations are marked in bold (*r* > 0.5 and *p* < 0.01)

^a^miR-34a, miR-320b and miR-378a were previously shown to increase with aging; miR-20a, miR-30b, miR-106b, miR-191, miR-301a and miR-374a were previously shown to decrease with aging

In a next step, we compared both patient groups to determine whether they also showed differences with respect to aging biomarkers and/or clinical variables at inclusion. Interestingly, patients from cluster A indeed exhibited significantly higher fTRST, IL-6, TNFα and MCP-1 (Table 6), along with a higher mean age. LOFS was also apparently decreased in these patients (mean LOFS were 6.9 and 7.7 for clusters A and B, respectively), but this difference was not statistically significant (Table 6). Moreover, cluster A patients showed a markedly higher tendency to experience a decline in QoL during chemotherapy: 31.9% of cluster A patients, versus only 7.4% of cluster B patients, scored lower on QoL at 3 months (i.e. at the end of chemotherapy treatment) as compared to inclusion (*p* = 0.051).

**Table 6:** Differences in biological and clinical aging markers between miRNA-based patient clusters at inclusion

Aging/frailty parameter	Statistic	Patient cluster A	Patient cluster B	*p*-value
Calendar age	N	43	45	
	Mean ± SD	76.5 ± 5.2	73.8 ± 3.1	**0.018**
BALDUCCI				
Fit	n/N (%)	9/43 (20.9%)	12/44 (27.3%)	
Vulnerable	n/N (%)	12/43 (27.9%)	16/44 (36.4%)	
Frail	n/N (%)	22/43 (51.1%)	16/44 (36.4%)	0.378
LOFS	N	42	44	
	Mean ± SD	6.9 ± 2.2	7.7 ± 1.7	0.119
G8-total	N	43	45	
	Mean ± SD	14.1 ± 1.8	14.2 ± 2.1	0.571
fTRST	N	36	30	
	Mean ± SD	2.3 ± 1.0	1.8 ± 0.9	**0.046**
Telomere length (T/S ratio)	N	33	38	
	Mean ± SD	0.67 ± 0.17	0.74 ± 0.28	0.423
IL-6 ^*a*^ (pg/mL)	N	43	45	
	Median (IQR)	4.3 (2.4; 6.4)	1.7 (1.0; 3.3)	**< 0.001**
IL-10 (pg/mL)	N	41	43	
	Mean ± SD	0.26 ± 0.25	0.25 ± 0.30	0.528
IGF-1 (pg/mL)	N	43	45	
	Mean ± SD	73 ± 24	79 ± 24	0.308
TNFα (pg/mL)	N	43	45	
	Median (IQR)	1.5 (0.9; 2.3)	1.2 (0.7; 1.6)	**0.032**
MCP-1 (pg/mL)	N	43	45	
	Mean ± SD	197 ± 90	146 ± 57	**< 0.001**
RANTES (ng/mL)	N	43	45	
	Mean ± SD	52 ± 42	64 ± 47	0.179

^a^For IL-6 and TNFα, median and IQR was used instead of mean ± SD, to moderate the effect of outliers

*p*-values < 0.05 are marked in bold

## Discussion

We have recently published a scientific article reporting on the evolution of clinical and biological aging markers in older BC patients receiving chemotherapy [24]. This study demonstrated that adjuvant TC chemotherapy had basically no impact on aging and frailty during a one-year period; we only detected a modest and temporary alteration of clinical aging indicators, while established aging biomarkers such as IL-6 did not show significant fluctuations during the a one-year period.

Aiming to corroborate these findings with additional aging biomarkers, we decided to analyse, on the same patient cohort, a panel of 9 miRNAs that were clearly differentially expressed in elderly BC patients compared to their younger counterparts in a previous exploratory miRNA pilot study also carried out by our group [25]. This panel included miR-34a, miR-320b and miR378a, which were previously found to be positively correlated with age, and miR-20a, miR-30b, miR-106b, miR-191, miR-301a and miR-374a, which previously showed negative associations with age.

We have found that several of these ‘aging miRNAs’ changed during the course of the study, but these changes seemed rather transient and, most importantly, no striking differences in miRNA evolution between CTG and CG were revealed. Although an apparent discrepancy between CTG and CG was noted for miR-106b, the relevance of this observation can be questioned since the statistical significance was only modestly persuasive and the direction of the change is not in line with logic expectancies: given the previously established negative correlation of miR-106b with age, one would rather expect a decrease of miR-106b in chemotherapy-treated patients. Thus, the present study basically supports our previous statement that chemotherapy treatment is not associated with marked acceleration of the aging process in elderly BC patients.

Besides this primary research question, we have also further explored the relationships existing between the 9 selected ‘aging miRNAs’ and other parameters reflecting the patient’s clinical and biological age. In line with our previous findings, the present results show a decrease of miR-301a with calendar age at inclusion. Four other miRNAs of the panel, namely miR-30b, miR-106b, miR-320b and miR-374a-5p, showed trends consistent with our pilot data, but were not retained as independent explanatory variables for age in a multivariable model, probably because of the small age range of this study cohort (all ≥70).

Of relevance, miR-320b and miR-374a, which we previously found to be associated only with chronological age, now showed a clear correlation with frailty (LOFS) as well. Frail patients, scoring lower on the LOFS scale, exhibited higher miR-320b and lower miR-374a levels, as could be anticipated from the respectively positive and negative correlations of these miRNAs with age. Thus, these two miRNAs that evolve oppositely with aging are actually also oppositely correlated with LOFS. It should be noted that the basic Balducci frailty score, unlike the more refined LOFS, did not show significant associations with any of the 9 miRNAs in multivariable analysis. For the G8 screening tool, scored at inclusion and yielding a lower score with increasing frailty, 4 miRNAs of the aging panel (i.e. miR-106b, miR-191, miR-320b and miR-374a) were identified as independent predictors in the multivariable model selection. This finding should, however, be interpreted with caution, since 2 of these miRNAs (miR-106a and miR-191) were correlated in the opposite direction as expected and none of the four miRNAs showed a significant correlation with G8 score in univariable analysis. For the fTRST, yielding a higher score with increasing frailty, we found a consistent negative association with miR-301a, which is in full agreement with the age-related decrease of this miRNA [25]. Thus, nearly all associations found are consistent with the known age-related trends. Overall, these data are suggestive of a link between the aging miRNAs and clinical frailty, whereas our previous miRNA study only uncovered strong associations of the 9 miRNAs with chronological age but not with frailty [25]. The larger number of patients included in the present study probably afforded a greater statistical power, allowing detection of biological/clinical correlations that remained unrevealed in our initial miRNA pilot study.

With regard to the correlations between the aging miRNA panel and other biomarkers of aging, 6 of the selected miRNAs (though 3 of them failed to reach statistical significance in the multivariable analysis), correlated with circulating levels of IL-6, a well-known aging biomarker [24] that we previously also found to be predictive of frailty [5]. Moreover, lower mean leukocyte telomere length, a well-described hallmark of biological aging, and increased plasma TNFα were both associated with higher miR-320b levels. This is once more in accordance with the age-related increase in miR-320b, as previously reported [25]. Finally, the plasma inflammatory chemokine MCP-1, previously reported to increase with aging [5], was positively correlated with miR-378a, which also increases with age, and negatively correlated with miR-301a, which decreases with age.

When studying interconnections between the 9 miRNAs, we found that patients actually clustered into two major distinct subgroups with different ‘aging miRNA’ expression profiles: patients from one cluster showed significantly lower expression of miR-30b, miR-191, miR-301a and miR-374a and higher expression of miR-378a, as compared to the other cluster, which is consistent with an ‘older’ miRNA profile. In perfect agreement, these patients exhibited increased IL-6, TNFα, MCP-1 and also significantly higher fTRST and a markedly higher propensity towards decline in functionality during chemotherapy treatment. Apparently, aging miRNA-based patient clustering actually goes along with signs of different biological and clinical age between clusters.

Taken together, our findings point to marked correlations of (1°) chronological age with miR-301a, (2°) biomarkers of aging with miR-106b, miR-301a, miR-320b, miR-374a and miR-378a, and (3°) clinical frailty with miR-320b and miR-374a. Experiments on mice have previously suggested that miR-320b may be involved in cardiac degeneration due to hyperglycemia, as high levels of this miRNA are found in the diabetic heart [47]. For miR-374a, however, this is the first study, to the best of our knowledge, that reveals an association with aging/frailty. We understand that our finding is limited by its correlative nature, so that further research is needed to assess whether a causative relationship links these particular miRNAs and physical involution.

Some miRNAs previously found over- or under-expressed with age, like miR-301a, or also with aging-related health conditions (such as miR-34a and miR-20a) [25, 26, 48] confirmed a trend in the same direction in our present study, albeit without reaching a formal statistical significance. One possible explanation could be that there is no actual association between the examined variables. However, since we are studying aging miRNAs in the context of elderly BC patients, we also have to bear in mind the multifaceted nature of the neoplastic process: indeed several miRNAs overexpressed during aging are underexpressed in patients affected by neoplasms or vice versa. In fact, an association with cancer opposite to that observed during aging has previously been demonstrated for miR-34a, miR-106b, miR-191-5p, miR-301a, although some studies generated conflicting results [29, 32, 34, 37–41, 49]. In particular, experiments on murine cell cultures revealed that miR-34a, showing age-related increase, acts as an oncosuppressor molecule within the p-53 regulatory pathway [41]. Evidence of reduced levels of this miRNA in hepatocellular carcinoma samples suggests that these functions may be exerted in humans as well [35, 50]. On the other hand, miR-106b and miR-20b, which both decrease at higher age, belong to the so-called onco-miR cluster 17~ 92 and are known to promote cancer growth by different mechanisms, including the escape of senescence [28, 33, 36, 38, 40]. In particular, miR-106b, which promotes cell cycle progression by targeting the regulatory protein p21, is upregulated both in cancer tissue and plasma of BC patients, and may be responsible of chemoresistance in BC cell cultures [40].

Moreover, several mechanisms and molecular pathways link cancer and aging in a not straightforwardly understandable way. Aging is a multifactorial process, in which a major role is played by senescent cells. Cellular senescence is a protective anti-cancer mechanism at young age, arresting the cell cycle in case of acquired irreversible DNA damage. However, the drawbacks of an accumulation of senescent cells, most particularly the altered secretion of inflammatory mediators, are mirrored at higher age by nervous, cardiovascular, immune-endocrine degeneration and a higher susceptibility to cancer itself. Based on all these data, we cannot exclude a confounding role of the oncological pathology itself. It is thus important to study aging biomarkers specifically in cancer populations, and not only in non-cancer older populations.

The lack of convincing statistical significance for certain ‘aging miRNAs’ towards particular aging/frailty-related response variables may also be explained by the notion that several miRNAs may act in a weak but subtly coordinated fashion scarcely appreciable by conventional statistics. As a consequence, we were not surprised to observe general trends in the directions we had foreseen, yet without individual miRNAs standing out as ‘the ultimate’ aging/frailty-related miRNA(s). What we found is rather an intricate network of weakly acting miRNAs; in fact each of the 9 miRNAs from our ‘aging miRNA panel’ somehow showed significant, or at least borderline, association(s) with one or more of the biological and/or clinical aging/frailty markers we previously evaluated [24], indicating that our ‘aging miRNA’ panel indeed consists of miRNAs that are truly relevant in the context of the aging process.

Of course, as stated previously, this study has its limitations due to the intrinsic peculiarities and heterogeneity of this particular oncogeriatric population, and should be considered as a starting point towards a thorough understanding of the complex relationships between cancer, aging and chemotherapy.

## Conclusion

In conclusion, the uppermost result of this study is the absence of any difference in the serum aging miRNA profile between chemotherapy-treated and control patients during and after adjuvant chemotherapy. This confirms our earlier findings with regard to the lack of a major impact of chemotherapy on a variety of biological and clinical aging markers.

Moreover, we created a miRNA-based clustering model resulting into two distinct groups exhibiting significantly different results for several biological/clinical aging parameters.

Although this is only a beginning, gaining knowledge about the interaction between the aging process and cancer treatment is a crucial step in the process of tailoring our treatments to older patients.

## Abbreviations

BC: Breast cancer
CG: Control group
CTG: Chemo group
GA: Geriatric assessment
IL-6: Interleukin-6
MCP-1: Monocyte Chemoattractant Protein-1
miRNA(s): MicroRNA(s)
TC: Docetaxel and cyclophosphamide
TNFα: Tumor Necrosis Factor-alpha
IL-10: Interleukin-10
RANTES: Regulated on Activation, Normal T-Cell Expressed and Secreted
CCL5: C-C motif chemokine-5
CCL2: C-C motif chemokine-2
IGF-1: Insulin-like Growth Factor-1
SIRT-1: Sirtuin 1
G-CSF: Granulocyte-colony stimulating factor
NCCN: National Comprehensive Cancer Network
Her2: Human epidermal growth factor receptor 2
fTRST: Flemish version of the Triage Risk Screening Tool
LOFS: Leuven Oncogeriatric Frailty Score
ECOG-PS: Eastern Cooperative Oncology Group – Performance Status
QoL: Quality of Life
